# Pharmacogenomic analysis of retinoic-acid induced dyslipidemia in congenic rat model

**DOI:** 10.1186/1476-511X-13-172

**Published:** 2014-11-17

**Authors:** Michaela Krupková, František Liška, Lucie Šedová, Drahomíra Křenová, Vladimír Křen, Ondřej Šeda

**Affiliations:** Institute of Biology and Medical Genetics, the First Faculty of Medicine, Charles University and the General Teaching Hospital, Albertov 4, 12800 Prague, Czech Republic; Institute of Molecular Genetics of the Academy of Sciences of the Czech Republic, Vídeňská 1083, 142 20 Prague 4, Czech Republic

**Keywords:** Pharmacogenomics, Metabolic syndrome, Animal model, Muscle transcriptome, All-trans retinoic acid, *Zbtb16*

## Abstract

**Background:**

All-trans retinoic acid (ATRA, tretinoin) is a vitamin A derivative commonly used in the treatment of diverse conditions ranging from cancer to acne. In a fraction of predisposed individuals, the administration of ATRA is accompanied by variety of adverse metabolic effects, particularly by the induction of hyperlipidemia. We have previously derived a minimal congenic SHR.PD-(*D8Rat42-D8Arb23*)/Cub (SHR-*Lx*) strain sensitive to ATRA-induced increase of triacylglycerols and cholesterol under condition of high-sucrose diet. SHR-*Lx* differs only by 7 genes of polydactylous rat (PD/Cub) origin from its spontaneously hypertensive rat (SHR) progenitor strain.

**Methods:**

Adult male rats of SHR and SHR-*Lx* strains were fed standard diet (STD) and experimental groups were subsequently treated with ATRA (15 mg/kg) via oral gavage for 16 days, while still on STD. We contrasted the metabolic profiles (including free fatty acids, triacylglycerols (TG) and cholesterol (C) in 20 lipoprotein fractions) between SHR and SHR-*Lx* under conditions of standard diet and standard diet + ATRA. We performed transcriptomic analysis of muscle tissue (m. soleus) in all groups using Affymetrix GeneChip Rat Gene 2.0 ST Arrays followed by Ingenuity Pathway Analysis and real-time PCR validation.

**Results:**

In response to ATRA, SHR-*Lx* reacted with substantially greater rise in TG and C concentrations throughout the lipoprotein spectrum (two-way ANOVA strain * RA interaction significant for C content in chylomicrons (CM), VLDL and LDL as well as total, CM and HDL-TG).

**Conclusions:**

According to our modeling of metabolic and signalization pathways using differentially expressed genes we have identified a network with major nodes (including *Sirt3, Il1b, Cpt1b* and *Pparg*) likely to underlie the observed strain specific response to ATRA.

**Electronic supplementary material:**

The online version of this article (doi:10.1186/1476-511X-13-172) contains supplementary material, which is available to authorized users.

## Background

All-trans-retinoic acid (ATRA, tretinoin) is a vitamin A derivative that serves as a prominent hub of many signaling and metabolic pathways involved in range of processes from development to energy homeostasis [1]. Both excess and shortage of ATRA were shown to have pathophysiological consequences and rather tight maintenance of ATRA levels is necessary both in development and adult life in order to prevent disease manifestation [2]. Apart from its well described role as a teratogen, variety of side-effects associated with ATRA therapy in postnatal stages of life have been described both for topical [3] and systemic administration [4]. Serum concentrations of lipids [5, 6], glucose tolerance or bone metabolism [7] are among the systems most frequently affected in adverse reactions to ATRA administration. On the other hand, several recent studies suggested the potential of tretinoin and other, more specific retinoid receptor agonists in treatment of metabolic syndrome or its components [8, 9]. We have previously established a congenic rat strain SHR.PD(*D8Rat42-D8Arb23*)/Cub (SHR-*Lx* hereafter) particularly sensitive to teratogenic actions of ATRA [10] as well as ATRA-induced dyslipidemia when fed high-sucrose diet [11]. The SHR-*Lx* is also insulin resistant compared to its spontaneously hypertensive rat (SHR) progenitor [12], from which it differs in a limited region of chromosome 8. While in the original work we assigned 14 protein-coding genes to the differential segment present in SHR-*Lx* [12], we have recently fine mapped its span to less than 800 kb encompassing only seven genes [13]. In the current study, we have investigated whether the sensitivity of SHR-*Lx* to ATRA-induced dyslipidemia is present also under standard diet condition. At the same time, we explored whether the pharmacogenetic interaction of RA and the differential segment is reflected in the transcriptome of skeletal muscle, the tissue shown to be metabolically challenged in the SHR-*Lx* [12].

## Results

### Morphometric profile

When fed standard diet, both strains showed comparable body weight and relative weights of visceral and retroperitoneal adipose tissue depots. The liver and kidney weights were lower in SHR-*Lx* congenic, on the contrary, weight of adrenals was increased in comparison to SHR (Table 1). The 16-day RA administration did not result in changes of total body weight (repeated measures ANOVA was insignificant for Strain, ATRA and Strain* ATRA) or adipose tissue depots in either of the strains. The ATRA-treated SHR showed significantly higher relative weights of liver, heart and kidneys compared to ATRA-treated SHR-*Lx*. The only morphometric effect attributable to RA was increase of liver weight per 100 g body weight exclusively in SHR (Table 1). The fasting glycemia did not vary significantly regardless of strain or ATRA administration status.

**Table 1:** **Morphometric comparison of SHR vs. SHR-** ***Lx*** **rats**

Trait	Standard diet		Standard diet + ATRA	
	SHR	SHR-***Lx***	SHR	SHR-***Lx***
	(n = 7)	(n = 7)	(n = 7)	(n = 8)
Body weight, g	294 ± 6	275 ± 8	284 ± 7	265 ± 10
Liver, g/100 g b.wt.	3.73 ± 0.08	3.53 ± 0.05^a^	4.04 ± 0.09†	3.72 ± 0.03^b^
Heart, g/100 g b.wt.	0.40 ± 0.01	0.40 ± 0.02	0.41 ± 0.01	0.37 ± 0.01^a^
Kidney, g/100 g b.wt.	0.711 ± 0.003	0.65 ± 0.01^c^	0.71 ± 0.01	0.66 ± 0.01^b^
Adrenals, mg/100 g b.wt.	14.1 ± 0.3	15.7 ± 0.4^b^	14.4 ± 0.3	14.8 ± 0.4
EFP wt., g/100 g b.wt.	0.89 ± 0.03	0.84 ± 0.03	0.88 ± 0.03	0.83 ± 0.04
RFP wt., g/100 g b.wt.	1.05 ± 0.06	0.92 ± 0.05	1.02 ± 0.06	0.90 ± 0.06

Morphometric profile of SHR vs. SHR-*Lx* rats. The significance levels are indicated as follows: ^a, b, c^…p <0.05 and 0.01 and 0.001, respectively for differences between SHR and SHR-*Lx* under conditions of a single diet; †…p <0.01, respectively, for RA effect within individual strain. Values are shown as mean ± S.E.M.; b.wt….body weight; EFP…epididymal fat pad; RFP…retroperitoneal fat pad.

### Detailed lipid profile

**Figure 1:**
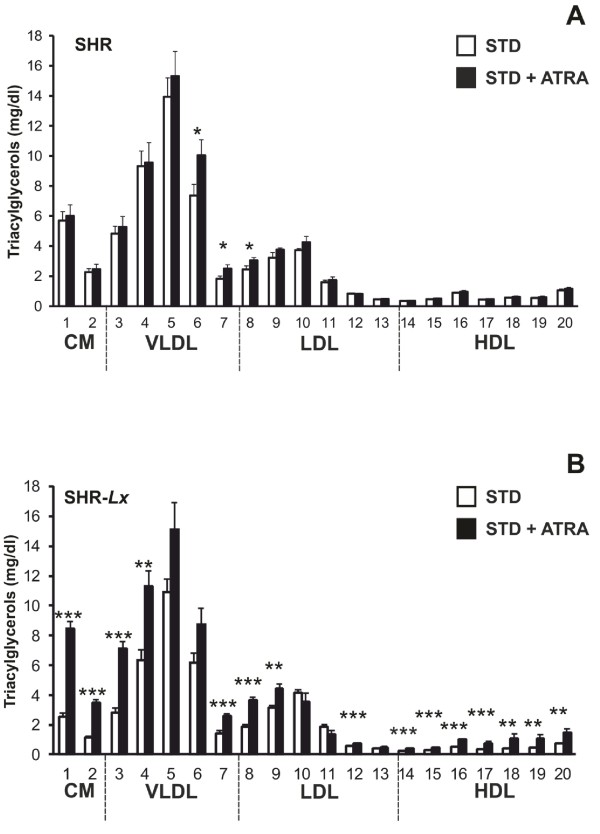
**The triacylglycerol content in 20 lipoprotein subfractions in standard diet-fed (STD, open symbols) and ATRA-treated (STD + ATRA, closed symbols) SHR (panel A) vs. SHR-** ***Lx*** (panel B) male rats (n = 7-8 / strain* treatment). Within the graph, the significance levels of strain comparison (SHR vs. SHR-*Lx*) by post-hoc Tukey's honest significance difference test of the two-way ANOVA with STRAIN and ATRA as major factors are indicated as follows: *…p < 0.05; **…p < 0.01; ***…p < 0.001. The allocation of individual lipoprotein subfractions to major lipoprotein classes is shown in order of particle’s decreasing size from left to right. CM…chylomicron, VLDL…very low-density lipoprotein, LDL…low-density lipoprotein, HDL…high-density lipoprotein.

**Figure 2:**
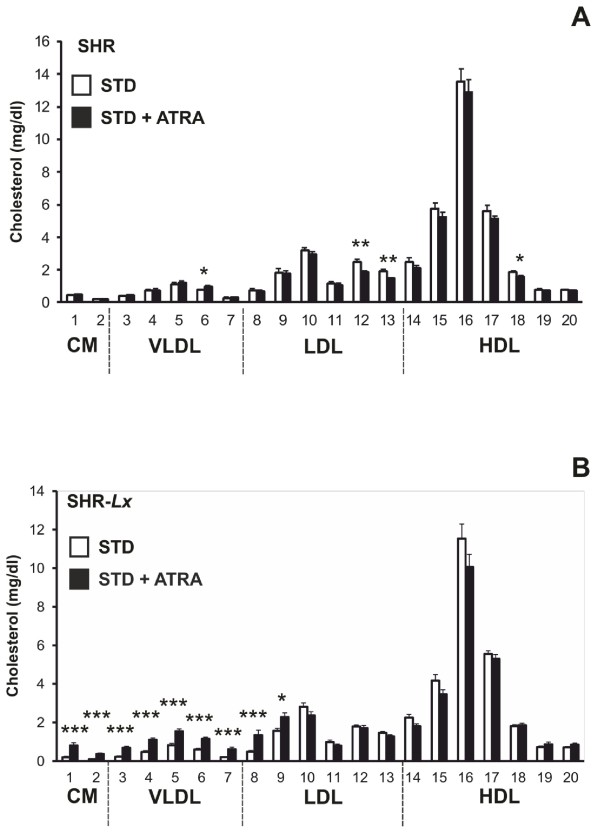
**The cholesterol content in 20 lipoprotein subfractions in standard diet-fed (STD, open symbols) and ATRA-treated (STD + ATRA, closed symbols) SHR (panel A) vs. SHR-** ***Lx*** (panel B) male rats (n =7-8 / strain* treatment). Within the graph, the significance levels of strain comparison (SHR vs. SHR-*Lx*) by post-hoc Tukey's honest significance difference test of the two-way ANOVA with STRAIN and ATRA as major factors are indicated as follows: *…p < 0.05; **…p < 0.01; ***…p < 0.001. The allocation of individual lipoprotein subfractions to major lipoprotein classes is shown in order of particle’s decreasing size from left to right. CM…chylomicron, VLDL…very low-density lipoprotein, LDL…low-density lipoprotein, HDL…high-density lipoprotein.

The control SHR rats had significantly higher total and chylomicron TG levels compared to STD-fed SHR-*Lx* (Table 2). The ATRA administration resulted in substantial increase of triacylglycerol concentrations in all major lipoprotein fractions except LDL exclusively in SHR-*Lx* (Table 2, Figure 1). As a result, chylomicron and VLDL-TG concentrations were higher in the ATRA-treated congenics compared to SHR (Table 2, Figure 1). We observed similar pattern for cholesterol distribution across the lipoprotein spectrum, reflected by significant Strain * ATRA interactions for chylomicron, VLDL and LDL cholesterol (Tables 2 and 3). So, while total cholesterol as well as cholesterol concentration in all major lipoprotein classes were significantly higher in control SHR rats compared to SHR-*Lx* controls, chylomicron and VLDL-C substantially increased only in SHR-*Lx* to levels significantly higher than those in SHR (Table 2, Figure 2). LDL-C actually slightly decreased only in ATRA-treated SHR, the HDL-C did not change in either of the strains and remained thus lower in the SHR-*Lx*. No differences were observed for fasting glycerol regardless of strain or ATRA status. The sizes of VLDL, LDL and HDL particles were similar in STD-fed animals of both strains. The treatment with ATRA induced increases of VLDL and LDL particle sizes together with a decrease of HDL size only in the SHR-*Lx*, resulting in their significant difference from the unchanged SHR values (Table 4).

**Table 2:** **Major triacylglycerol, cholesterol subfractions and free glycerol comparison between control and retinoic acid-fed SHR vs. SHR-** ***Lx*** **rats**

Trait (mg/dl)	Standard diet		Standard diet + ATRA	
	SHR	SHR-***Lx***	SHR	SHR-***Lx***
	(n = 7)	(n = 7)	(n = 7)	(n = 8)
**Triacylglycerol (TG)**				
Total TG	61.84 ± 4.50	46.33 ± 2.38^a^	69.87 ± 5.51	77.27 ± 4.66‡
Chylomicron TG	7.97 ± 0.83	3.66 ± 0.34^a^	8.47 ± 1.05	11.94 ± 0.67‡^,b^
VLDL-TG	37.27 ± 3.28	27.68 ± 2.05	42.67 ± 4.11	44.83 ± 4.37†^,a^
LDL-TG	12.29 ± 0.47	12.04 ± 0.39	14.07 ± 0.85	14.28 ± 1.12
HDL-TG	4.32 ± 0.13	2.95 ± 0.12	4.66 ± 0.36	6.23 ± 0.99‡
**Cholesterol (C)**				
Total C	45.92 ± 1.57	38.46 ± 1.55^c^	42.52 ± 1.34	40.26 ± 0.78
Chylomicron C	0.63 ± 0.05	0.29 ± 0.02^c^	0.69 ± 0.07	1.18 ± 0.20 ‡^,b^
VLDL-C	3.26 ± 0.27	2.32 ± 0.10 ^a^	3.70 ± 0.22	5.10 ± 0.46‡^,b^
LDL-C	11.26 ± 0.44	9.10 ± 0.31^c^	9.77 ± 0.37*	9.78 ± 0.39
HDL-C	30.77 ± 1.38	26.75 ± 1.37^a^	28.37 ± 1.20	24.20 ± 0.94^a^
**Glycerol**	1.07 ± 0.12	1.00 ± 0.06	1.24 ± 0.15	0.99 ± 0.02

Data are shown as mean ± S.E.M. The significance levels are indicated as follows: ^a, b, c^…p <0.05, 0.01 and 0.001, respectively for differences between SHR and SHR-*Lx* under conditions of a single diet; †, ‡… p <0.01 and 0.001, respectively, for RA effect within individual strain.

**Table 3:** **Two-way analysis of variance (ANOVA) results for morphometric and metabolic profile of SHR vs. SHR-** ***Lx*** **rats with STRAIN and RA as major factors**

Phenotype	STRAIN	ATRA	S*ATRA
Body weight	**0.023**	*0.23*	*0.97*
Liver, g/100 g b.wt.	**0.0005**	**0.0008**	*0.76*
Heart, g/100 g b.wt.	*0.13*	*0.47*	*0.10*
Kidney, g/100 g b.wt.	**<0.0001**	*0.60*	*0.77*
Adrenals, mg/100 g b.wt.	**0.010**	*0.38*	*0.12*
EFP wt., g/100 g b.wt.	**0.039**	*0.65*	*0.90*
RFP wt., g/100 g b.wt.	*0.13*	*0.64*	*0.96*
**Total C**	**0.0018**	*0.56*	*0.07*
Chylomicron C	*0.48*	**0.0003**	**0.001**
VLDL-C	*0.43*	**<0.0001**	**0.0007**
LDL-C	**0.011**	*0.31*	**0.010**
HDL-C	**0.0035**	*0.06*	*0.96*
**Total TG**	*0.37*	**0.0003**	**0.017**
Chylomicron TG	*0.59*	**<0.0001**	**<0.0001**
VLDL-TG	*0.31*	**0.0049**	*0.12*
LDL-TG	*0.97*	**0.017**	*0.77*
HDL-TG	*0.85*	**0.0030**	**0.013**
**Glycerol**	*0.13*	*0.41*	*0.37*
Fasting plasma glucose	*0.15*	*0.08*	*0.76*
**Lipoprotein particle size**			
VLDL-TG	0.61	*0.34*	**0.0075**
LDL-C	**0.0026**	**0.0013**	*0.07*
HDL-C	**0.0003**	**0.039**	*0.08*

The significance levels of two-way ANOVA’s STRAIN, RA and STRAIN*RA (S*RA) factor interactions are shown (significant p values in bold, non-significant in italics).

**Table 4:** **Lipoprotein particle size comparison control and retinoic acid-fed SHR vs. SHR-** ***Lx*** **rats**

Trait (nm)	Standard diet		Standard diet + ATRA	
	SHR	SHR-***Lx***	SHR	SHR-***Lx***
	(n = 7)	(n = 7)	(n = 7)	(n = 8)
VLDL-TG	49.51 ± 0.45	48.24 ± 0.49	48.48 ± 0.68	50.27 ± 0.41*^,a^
LDL-C	21.45 ± 0.25	21.54 ± 0.11	21.77 ± 0.12	22.52 ± 0.18‡^,b^
HDL-C	12.20 ± 0.04	12.10 ± 0.03	12.18 ± 0.03	11.93 ± 0.05‡^,c^

Data are shown as mean ± S.E.M. The significance levels are indicated as follows: ^a, b, c^…p <0.05, 0.01 and 0.001, for differences between SHR and SHR-*Lx* strains under conditions of a single diet; *, ‡… p <0.05 and 0.001, respectively, for RA effect within individual strain.

### Transcriptomic profile of skeletal muscle

**Figure 3:**
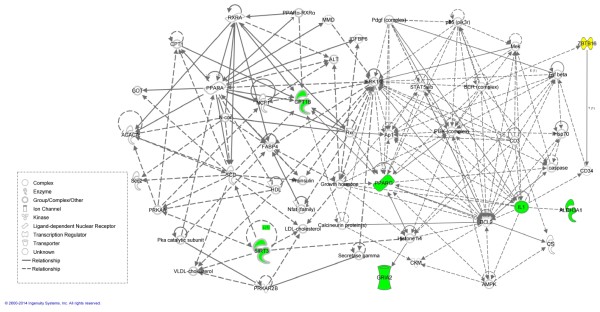
**Network analysis of pharmacogenetic interaction of RA.** The figure represents the network with highest score (IPA, Ingenutiy Systems) derived using the set of transcripts showing significant STRAIN (SHR, SHR-*Lx*) * ATRA (STD, STD + RA) interaction. The genes with validated expression changes by real-time PCR are shown in green. After *in silico* analysis of direct and indirect connections of the genes present within the chromosome 8 differential segment in SHR-*Lx* to the members of this highest-scoring network, only the *Zbtb16* gene (yellow) showed positive results.

In order to generate testable hypothesis on potential mechanism of the observed pharmacogenetic interaction, we have searched for distinct patterns of transcription changes in skeletal muscle. The canonical pathway enrichment showed only 4 significant results. The single pathway exclusively enriched in context of ATRA effect on SHR was *Ethanol degradation* (Benjamini-Hochberg (B-H) p = 6.88 × 10^-3^, including SHR-exclusive downregulation of *Aldh3a1*), the remaining three pathways were overrepresented only in context of ATRA effect on SHR-*Lx: D-myo-inostitol (1,4,5)-triphosphate degradation* (B-H p = 1.83 × 10^-3^), *Eicosanoid signaling* (B-H p = 3.81 × 10^-2^) and *Role of macrophages, fibroblasts and endothelial cells in rheumatoid arthritis* (B-H p = 1.83 × 10^-3^ including upregulation of *Il1b*). Next we generated networks based on the transcript sets showing the highest significance for STRAIN* ATRA pharmacogenetic interaction. The resulting network reaching the highest score is shown in Figure 3. In addition to retinoid receptors it contains and connects number of genes with known involvement in lipid metabolism, insulin resistance and inflammation. Then we explored the shortest possible connection of the identified network to all seven genes present in the differential segment of SHR-*Lx*. The only gene with identifiable connection to the network was *Zbtb16* (Figure 3). We have subsequently validated the microarray results using real-time PCR for several major transcripts showing distinct patterns the expression changes and at the same time representing major nodes identified by the network analysis (Additional file 1: Figure S1). In all cases we were able to corroborate the “direction” of the RA-induced effect, i.e. the relative up- or down-regulation of expression in the individual strain, yet the extent of the change seemed to be somewhat underestimated by the array.

## Discussion

Induction of hyperlipidemia belongs to one of the frequent side-effects of administration of retinoid receptor agonists including ATRA to humans. Hyperlipidemia actually belonged to the dose-limiting toxicity factor for oral treatment of oncological conditions with rexinoids (selective retinoic X receptor ligands), increasing particularly the triacylglycerols by hundreds of percent [14]. Even though the individual sensitivity towards the adverse metabolic effects of retinoids is most likely driven by particular pharmacogenetic interactions, there is very little information available on the genetic variants involved except for the treatment of several cancers including acute promyelocytic leukemia [15], neuroblastoma [16] or head and neck squamous cell carcinoma [17]. We have previously identified the mutant rat *Lx* allele as a genetic variant sensitizing to ATRA-mediated teratogenic effects [10, 18, 19]. During the process of positional cloning of *Lx* we were eventually able to trap the variant within a minimal differential segment in the SHR-*Lx* congenic strain [13, 20]. When fed high-sucrose diet, the adult SHR-*Lx* rats mimicked the hyperlipidemic reaction to ATRA exposure contrasting with its SHR progenitor [11]. In the current study, we show this oversensitivity to be preserved under conditions of standard diet feeding and thus not being dependent on diet* medication interaction(s). This seems to be in direct contrast with the reports from several recent studies in C57BL/6 mice. In this murine strain, ATRA was shown to prevent high-fat, high-sucrose diet-induced obesity [21] and ameliorate glucose intolerance, insulin resistance and even dyslipidemia [22, 23]. However, apart from possible species differences, the reason for such disparate results may as well lie in the genetically determined susceptibility itself – the SHR control strain in our study did not actually show any metabolic deterioration in response to ATRA, the LDL-C levels even slightly dropped. The SHR-*Lx* strain thus truly represents a model of enhanced susceptibility found in the humans manifesting substantial adverse effects to ATRA treatment. We have previously established that insulin-stimulated glucose utilization in muscle of SHR-*Lx* is impaired in comparison to SHR [12]. Given the importance of the skeletal muscle tissue for overall insulin sensitivity and lipid metabolism, we have assessed the transcriptomic correlates of the observed pharmacogenetic interaction in the *mm. solei* of both strains. The resulting network of transcripts central to strain-specific reaction to ATRA (Figure 3) corresponds to our biochemical findings and is suggestive concerning the possible mechanism of SHR-*Lx* sensitivity. One of the important nodes connecting several modules of the network is *Sirt3,* specifically downregulated by ATRA in SHR-*Lx* (Additional file 1: Figure 1). Downregulation of this gene was found in rodent models of diabetes and was reported to impair insulin signaling in the muscle and induce oxidative stress [24]; hence it was proposed as major regulator of metabolic flexibility of skeletal muscle [25]. It is through stearoyl-CoA desaturase (*Scd1*) that *Sirt3* connects to an apparent lipid-related module with peroxisome proliferator activated receptor alpha at its middle. The SHR-*Lx* congenic shows more than three- and twofold higher expression of *Scd1* in control and ATRA-treated animals when compared to SHR. As recently reviewed, increased *Scd1* expression in skeletal muscle (both in transgenic models and obese human subjects) is associated with decreased beta-oxidation, increased TG accumulation and muscle insulin resistance [26]. Within this lipid-related cluster, we have validated the pronounced downregulation of *Cpt1b* (a key enzyme in the control of fatty acid oxidation) in response to ATRA in SHR*-Lx.* Peroxisome proliferator activated receptor gamma represents the focal point of the whole network. Activated solely in SHR by ATRA, it provides a link between the metabolic and cell proliferation/apoptosis-related genes with evidence for strain-specific behavior in the current study. None of the 7 genes present in the differential segment of the congenic strain were distinctly up- or downregulated by ATRA in the soleus muscle. However, testing the existing paths between the 7 genes and the top mechanistic network using the Ingenuity Pathway Analysis revealed suggestive connection only for the *Zbtb16* gene. This transcriptional repressor is known to interact with retinoic acid signaling [27] and it plays important roles in numerous and diverse processes including, but not limited to differentiation of innate lymphocytes [28], stem cell renewal [29] and limb patterning and development [20, 30]. We have previously shown the *Lx* mutation present in the SHR-*Lx* congenic to result from 2.9 kb deletion of conserved noncoding element in *Zbtb16* gene and to be responsible for polydactyly as well as left ventricle hypertrophy, hypertension and insulin resistance [12, 13, 20] and to sensitize towards ATRA-induced teratogenicity [10]. In mice, complete knockout of *Zbtb16* [30] as well as spontaneous mutation (luxoid) [31] lead to infertility and number of skeletal patterning defects. However, mice carrying distinct missense *Zbtb16* mutation were fertile in spite of displaying hindlimb and axial skeleton abnormalities [32]. Very recently, hepatic knockdown of *Zbtb16* was shown to ameliorate hyperglycemia in *db/db* mice [33]. Unfortunately, no data on metabolic parameters or their sensitivity to ATRA or similar agents are available for any of the mutant *Zbtb16*-carrying mouse models. Since the only genetic difference between the two tested strains is confined to the chromosome 8 region harboring the seven abovementioned protein-coding genes, current data together with our previous results lead us to hypothesize that unique separation-of-function allele of *Zbtb16* in SHR-*Lx* may be responsible for the observed transcriptome shifts resulting in ATRA-induced hyperlipidemia in the SHR-*Lx* congenic model. It is possible that strain-specific changes of *Zbtb16*, *Htr3a, Htr3b, Usp28, Zw10, Tmprss5* or *Drd2* expression in other tissues (liver, adipose tissue) underlie the observed shift of transcriptomic and metabolic profiles and enhanced sensitivity of SHR-*Lx*. This belongs to one of the limitations of the current study and multi-tissue screen of histologically ascertained structural changes, gene expression variation and subsequent targeted functional studies will be necessary to fully resolve the mechanism between the observed strain-specific ATRA effects on lipid levels. Also, since the design of our study was cross-sectional utilizing a single dosing regimen in young adult rats to make the results comparable to the previous experiments involving high-sucrose diet with/without ATRA administration [11], it is possible that if older animals were used, the metabolic effects of ATRA might be pronounced to different extent. The modulating effect of aging on complex disease manifestation including gene-environment interactions has been described both in the SHR strain [34, 35] and the differential segment donor, the PD rat strain [36] previously. We have recently reported a significant difference of in systolic blood pressure between the two strains used in the current study, i.e. SHR and SHR-*Lx* [13]. During the radiotelemetric measurement, the diastolic pressure did not differ significantly between the strains neither under standard diet nor with a substantial salt load. As our study was focused on ATRA-induced dyslipidemia, we did not run a separate arm of the study to radiotelemetrically assess the blood pressure. We can expect that it would be necessary to significantly prolong the exposure of the rats to ATRA to observe the hemodynamic effect as e.g. in the study performed by Zhong et al. [37], where ATRA reduced the blood pressure of SHR rats only after 3-4 weeks of treatment.

## Conclusions

In summary, we have validated the SHR-*Lx* strain as a model of ATRA-induced hyperlipidemia and identified putative network reflecting the mechanism of the pharmacogenetic interaction.

## Methods

All experiments were performed in agreement with the Animal Protection Law of the Czech Republic which is in compliance with the European Community Council recommendations for the use of laboratory animals 86/609/ECC and were approved by the Ethical committee of the First Faculty of Medicine, Charles University in Prague.

### Rat strains

The spontaneously hypertensive rat (SHR/OlaIpcv, SHR hereafter, Rat Genome Database [38] RGD ID: 631848) was originally derived by recurrent selective breeding of Wistar rats by Okamoto and Aoki in Japan in 1963 [39]. The SHR.PD(*D8Rat42-D8Arb23*)/Cub (SHR-*Lx*, RGD ID: 1641851) congenic strain was derived by introgressing and subsequent narrowing down of the rat chromosome 8 differential segment of the polydactylous rat PD/Cub [18] origin into SHR genomic background as described previously [12, 13]. The *Lx* refers to the mutated “luxate” allele that originally arose in outbred Wistar rat colony and was subsequently fixed in PD/Cub strain [18]; recently, we showed that a deletion of deeply conserved noncoding element in *Plzf* (*Zbtb16*) is responsible for the affliction of the limb development in *Lx*-bearing animals [20].

### Experimental protocol

At all times, the animals had free access to food and water. Animals were held under temperature and humidity controlled conditions on 12 h/12 h light-dark cycle. Male SHR (n = 14) and SHR-*Lx* (n = 15) rats were fed standard laboratory chow ad libitum. At the age of 15 weeks, the rats were randomly split to experimental (n = 7-8/strain) and control (n = 7/strain) groups. All groups continued to be fed standard diet for 16 days, and at the same time, the experimental group was administered ATRA (15 mg/kg/day via oral gavage) while the control group received only vehicle via oral gavage. The food consumption, total body weight and non-fasting glycaemia were followed daily during this period. The rats were sacrificed in postprandial state and the weights of heart, aorta, liver, kidneys, adrenals, soleus muscle, epididymal and retroperitoneal fat pads were determined and the soleus muscle was snap frozen in liquid nitrogen for further analyses of gene expression.

### Metabolic measurements

The fasting glycemia was assessed after overnight fasting at day 14 of ATRA or vehicle administration. Blood for glycaemia determination (Ascensia Elite Blood Glucose Meter; Bayer HealthCare, Mishawaka, IN, validated by Institute of Clinical Biochemistry and Laboratory Diagnostics of the First Faculty of Medicine) was drawn from thetail incision. . Plasma lipoproteins were analyzed by an on-line dual enzymatic method for simultaneous quantification of cholesterol, triacylglycerol and free glycerol by HPLC at Skylight Biotech Inc. (Akita, Japan) according to the procedure described previously [11, 40].

#### Transcriptomic profiling and quantitative real-time PCR

Total RNA was extracted with TRIzol® reagent (Invitrogen, Carlsbad, CA) and purified with the RNeasy® MinElute cleanup kit (Qiagen, Valencia, CA) following the manufacturer’s recommendations. The quality of the total RNA was evaluated on an Agilent 2100 Bioanalyzer system (Agilent, Palo Alto, CA). Microarray experiments were performed using the Affymetrix GeneChip® Rat Gene 2.0 ST Arrays (Affymetrix, Santa Clara, CA) according to manufacturer’s instructions in triplicate for each strain* treatment combination (i.e. 12 arrays in total). The whole hybridization procedure was performed using the Affymetrix GeneChip® system according to the protocol recommended by the manufacturer. The hybridization was evaluated with Affymetrix GeneChip® Command Console Software and quality of the chips with Affymetrix Expression Console. Partek Genomics Suite 6.6 (Partek, St. Louis, Missouri) was used for subsequent data analysis. The data was normalized by Robust Multichip Average algorithm, which uses background adjustment, quantile normalization and summarization. To validate microarray gene expression data, quantitative real-time PCR (TaqMan) was used. Total RNA (2 μg) was reverse-transcribed with oligo-dT primers using the SuperScript III (Invitrogen). The following sets of Taqman probes (Applied Biosystems) were used: sirtuin 3 (*Sirt3*): Rn01501410_m1, mitochondrial elongation factor 2 (*Mief2*, *Smcr7*): Rn01495681_g1, interleukin 1 beta (*Il1b*): Rn00580432_m1, carnitine palmitoyltransferase 1b, muscle (*Cpt1b*): Rn00682395_m1, glutamate receptor, ionotropic, AMPA 2 (*Gria2*): Rn00568514_m1, peroxisome proliferator-activated receptor gamma (*Pparg*): Rn00440945_m1, aldehyde dehydrogenase 3 family, member A1 (*Aldh3a1*): Rn00694669_m1, retinol dehydrogenase 11 (all-trans/9-cis/11-cis) (*Rdh11*): Rn01499137_m1. Real-time PCR reaction was performed in triplicate with TaqMan® Gene Expression Master Mix (Applied Biosystems) according to the manufacturer’s protocol (Invitrogen) using Applied Biosystems 7000 Real-Time PCR System. Results were analyzed using the Livak analysis method [41] with glyceraldehyde 3-phosphate dehydrogenase as reference gene. Pathway and network analysis including assessment of overrepresentation of differentially expressed probes in canonical, metabolic and signaling pathways and ontological classes, common regulator effects and *in silico* network construction was performed using the Ingenuity Pathways Analysis software (spring 2014 version).

### Statistical analysis

All statistical analyses were performed using STATISTICA 10 CZ. When comparing morphometric, biochemical and transcriptomic variables, two-way ANOVA was used with STRAIN (SHR; SHR-*Lx*) and ATRA [STD; STD + ATRA] as major factors (Table 3 and Additional file 2: Table S1), followed in case of biochemical and morphometric data by post-hoc Tukey's honest significance difference test for comparison of the specific pairs of variables. For series of daily repeated measurements (body weight, food consumption, satient glycaemia), repeated-measures ANOVA was used. Functional analyses and canonical pathways analyses were performed using Benjamini-Hochberg multiple testing correction. Null hypothesis was rejected whenever the corrected p <0.05.

## Electronic supplementary material

Additional file 1: Figure S1: Validation of microarray results using qPCR. Fold changes are indicated (in log2) for ATRA effect in SHR strains (microarray: white bars; qPCR: red bars) and the SHR-*Lx* congenic (microarray: black bars; qPCR: green bars). *Sirt3*: sirtuin 3; *Smcr7*: mitochondrial elongation factor 2 (Mief2); *Il1b*: interleukin 1 beta; *Cpt1b*: carnitine palmitoyltransferase 1b, muscle; *Gria2*: glutamate receptor, ionotropic, AMPA 2; *Pparg*: peroxisome proliferator-activated receptor gamma; *Aldh3a1*: aldehyde dehydrogenase 3 family, member A1; *Rdh11*: retinol dehydrogenase 11 (all-trans/9-cis/11-cis). (PDF 69 KB)

Additional file 2: Table S1: Two-way ANOVA for triacylglycerol, cholesterol concentrations in 20 lipid subfractions (F1-F20) and lipoprotein particle size. (PDF 279 KB)
