# Supplementary figures and images for: Pharmacogenomic analysis of retinoic-acid induced dyslipidemia in congenic rat model
Source: Lipids Health Dis. 2014 Nov 17;13:172. doi: 10.1186/1476-511X-13-172 (PMC4247747; doi:10.1186/1476-511X-13-172)

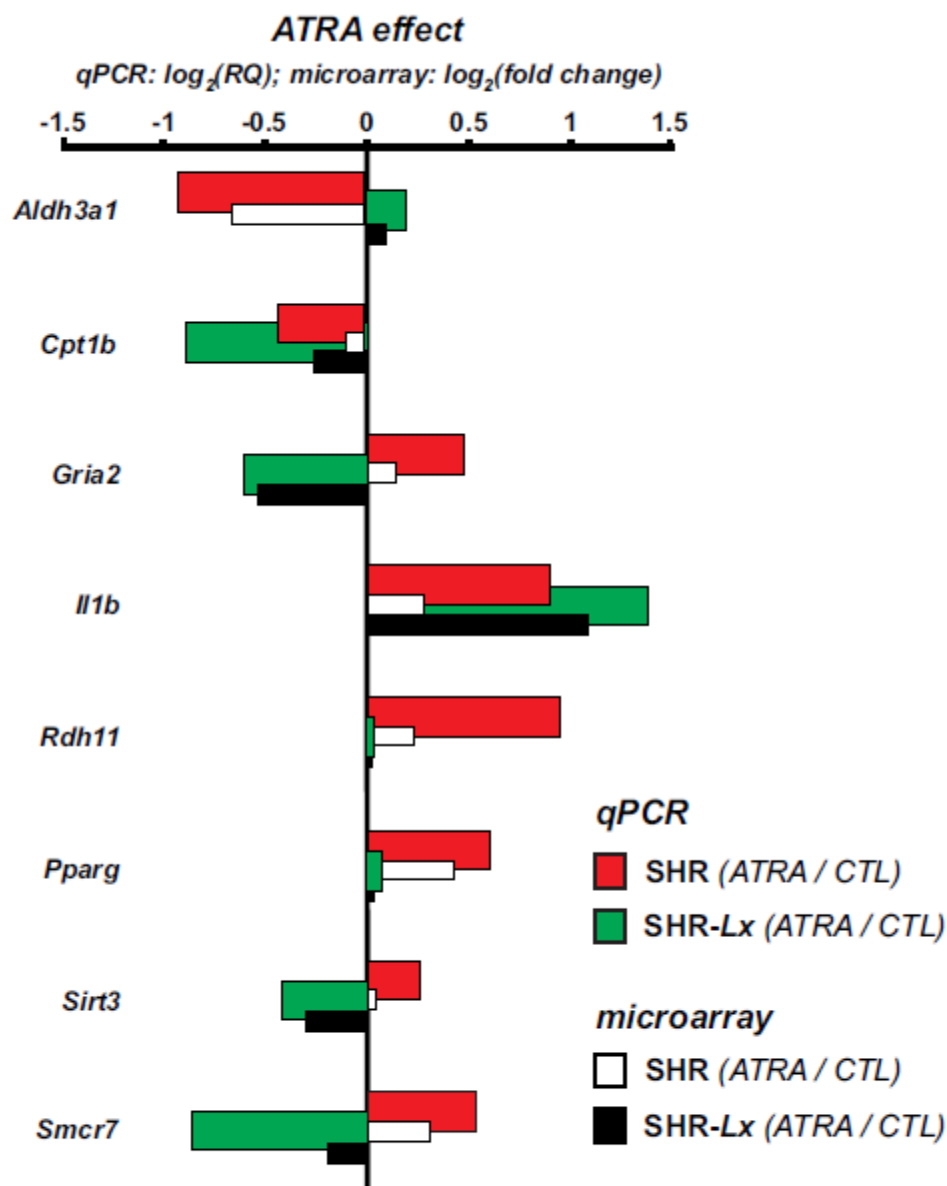

Additional file 1: Figure S1

Supplement: Supplementary file 1 — Additional file 1: Figure S1: Validation of microarray results using qPCR. Fold changes are indicated (in log2) for ATRA effect in SHR strains (microarray: white bars; qPCR: red bars) and the SHR-Lx congenic (microarray: black bars; qPCR: green bars). Sirt3: sirtuin 3; Smcr7: mitochondrial elongation factor 2 (Mief2); Il1b: interleukin 1 beta; Cpt1b: carnitine palmitoyltransferase 1b, muscle; Gria2: glutamate receptor, ionotropic, AMPA 2; Pparg: peroxisome proliferator-activated receptor gamma; Aldh3a1: aldehyde dehydrogenase 3 family, member A1; Rdh11: retinol dehydrogenase 11 (all-trans/9-cis/11-cis). (PDF 69 KB) [file 12944_2014_1160_MOESM1_ESM.pdf]
