# Supplementary material for: Pharmacogenomic analysis of retinoic-acid induced dyslipidemia in congenic rat model
Source: Lipids Health Dis. 2014 Nov 17;13:172. doi: 10.1186/1476-511X-13-172 (PMC4247747; doi:10.1186/1476-511X-13-172)
Supplement: Supplementary file 2 — Additional file 2: Table S1: Two-way ANOVA for triacylglycerol, cholesterol concentrations in 20 lipid subfractions (F1-F20) and lipoprotein particle size. (PDF 279 KB) [file 12944_2014_1160_MOESM2_ESM.pdf]

SUPPLEMENTARY INFORMATION

for manuscript

Pharmacogenomic analysis of retinoic-acid induced dyslipidemia in congenic rat model

Michaela Krupková[1], František Liška[1], Lucie Šedová[1], Drahomíra Křenová[1], Vladimír Křen[1], Ondřej Šeda[1,2]

[1]Institute of Biology and Medical Genetics, the First Faculty of Medicine, Charles University and the General Teaching Hospital, Prague, Czech Republic

[2]Institute of Molecular Genetics of the Academy of Sciences of the Czech Republic, Vídeňská 1083, 142 20 Prague 4, Czech Republic

Address for correspondence:

Ondřej Šeda, MD, PhD

Institute of Biology and Medical Genetics, First Faculty of Medicine,

Charles University in Prague,

Albertov 4, 12800 Prague 2, Czech Republic

Phone: (4202) 2496 8147; Fax: (4202) 2491 8666; email: oseda@lf1.cuni.cz

Supplementary Table. Two-way ANOVA for triacylglycerol, cholesterol concentrations in 20 lipid subfractions (F1-F20) and lipoprotein particle size.

**Phenotype**	**STRAIN**	**ATRA**	**S*ATRA**
**Triacylglycerol (TG)**			
F1 (CM)	*0.52*	**<0.0001**	**<0.0001**
F2 (CM)	*0.80*	**<0.0001**	**0.0002**
F3 (large VLDL)	*0.88*	**0.0002**	**0.0012**
F4 (large VLDL)	*0.55*	**0.020**	**0.033**
F5 (large VLDL)	*0.28*	*0.07*	*0.35*
F6 (medium VLDL)	*0.18*	**0.008**	*0.95*
F7 (small VLDL)	*0.41*	**0.0001**	*0.24*
F8 (large LDL)	*0.91*	**<0.0001**	**0.0044**
F9 (medium LDL)	*0.27*	**0.0031**	*0.19*
F10 (small LDL)	*0.71*	*0.91*	*0.13*
F11 (very small LDL)	*0.83*	*0.39*	*0.13*
F12 (very small LDL)	**<0.0001**	**0.016**	**0.003**
F13 (very small LDL)	*0.98*	*0.22*	*0.44*
F14 (very large HDL)	*0.06*	**<0.0001**	**<0.0001**
F15 (very large HDL)	**0.0015**	**0.0015**	**0.038**
F16 (large HDL)	**0.019**	**0.0003**	**0.004**
F17 (medium HDL)	*0.13*	**0.0016**	**0.003**
F18 (small HDL)	*0.39*	**0.027**	*0.06*
F19 (very small HDL)	*0.19*	**0.023**	**0.047**
F20 (very small HDL)	*0.93*	**0.008**	**0.041**

**Phenotype**	**STRAIN**	**ATRA**	**S*ATRA**
**Cholesterol (C)**			
F1 (CM)	*0.59*	**0.0004**	**0.0013**
F2 (CM)	*0.28*	**0.0001**	**0.0004**
F3 (large VLDL)	*0.28*	**<0.0001**	**0.0002**
F4 (large VLDL)	*0.52*	**0.0002**	**0.0005**
F5 (large VLDL)	*0.79*	**0.0002**	**0.0031**
F6 (medium VLDL)	*0.97*	**<0.0001**	**0.019**
F7 (small VLDL)	*0.07*	**0.0012**	**0.0056**
F8 (large LDL)	*0.15*	**0.0078**	**0.0034**
F9 (medium LDL)	*0.56*	*0.09*	*0.06*
F10 (small LDL)	**0.016**	*0.06*	*0.55*
F11 (very small LDL)	**0.040**	*0.17*	*0.67*
F12 (very small LDL)	**0.006**	**0.014**	*0.06*
F13 (very small LDL)	**0.0031**	**0.0035**	*0.18*
F14 (very large HDL)	*0.18*	**0.0031**	*0.94*
F15 (very large HDL)	**<0.0001**	*0.07*	*0.72*
F16 (large HDL)	**0.0027**	*0.15*	*0.57*
F17 (medium HDL)	*0.84*	*0.18*	*0.70*
F18 (small HDL)	*0.13*	*0.13*	**0.031**
F19 (very small HDL)	*0.57*	*0.66*	*0.23*
F20 (very small HDL)	*0.47*	*0.27*	*0.13*

The significance levels of two-way ANOVA's STRAIN, ATRA and STRAIN*ATRA (S*ATRA) factor interactions are shown (significant p values in bold, non-significant in italics).
